# Supplementary material for: Validation of reference genes for gene expression studies in tartary buckwheat (Fagopyrum tataricum Gaertn.) using quantitative real-time PCR
Source: PeerJ. 2019 Feb 26;7:e6522. doi: 10.7717/peerj.6522 (PMC6396815; doi:10.7717/peerj.6522)
Supplement: Supplemental Information 1 [file peerj-07-6522-s001.docx]

qRT-PCR primers are capitalized and underlined.

***FtDFR***

NCBI Genbank ID: GU169468.1

ATGGTTGCTGAGGGAGAGATCGTCTGTGTCACCGGCGCTTCCGGCTTCGTCGGCTCATGGCTCGTCATGAGGCTCCTCGAACATGGCTACGTTGTCCGTGCCACCGTCAGGGATCCAAGCAACATGAAGAAAGTGAAGCACTTGTTGGATTTGCCCAAATCGAAGACGAATTTGAGCCTCTGGAAAGCCGATCTCAGTGAAGAAGGAAGCTTTGACGAAGCAATTCAAGGCTGTGCTGGTGTTTTCCATGTTGCGACTCCCATGGATTTCGAGTCCAAGGATCCTGAGAATGAGGTGATTAAGCCAACCATCAATGGTATGCTGGACATCATGAAAGCATGCCTGAAGGCGAATGTGCGGAAATTGGTGTTCACATCTTCAGCCGGAACGGTCAACGTTGAAGAGAAACAAAAGCCTGTGTACGATGAGACTTGCTGGAGTGACGTTGACTTCTGCCGAAGAGTTAAGATGACTGGCTGGATGTACTTCGTATCCAAGACATTGGCAGAACAAGCAGCTTGGAAATTTGCTGAGGAAAACAACATGGATTTCATTAGCATTATCCCAACTCTCGTTGTCGGCCCTTTCATTATGCCAAGTTTCCCTCCAAGTCTCATCACAGCCCTCTCCCCAATCACAAGAACTGAGGGTCACTACACAATCATAAAACAATGTCAGTACGTACACTTGGACGATTTATGCATGTCTCACATTTACCTCTATGAGAAGGCTGGCTCGAAAGGACGTTACGTTTGTTCTTCCCACAATGCTACCATTTATGACCTCGGAAAAAT**GCTCCGAAACAAGTATCCCGA**GTACAATGTCCCTACCAAGTTTAGGGATTTTGATGAGAACATGGAAGCGGTGTCGTTCTCATCGAAGAAGCTGACCGATGAAGGGTTCGAGTTCAAGTATAGCTTGGAGGACA**TGTTTGTTGGTGCTGTGGAGAC**TTGTAGGGAGAAGGGCTTGCTTCCCAAAACCTTTGAGGAGATTGAGAAGAACCATGTAAATGGTAATGGCCATTGA

***FtH3***

NCBI Genbank ID: HM628903.1

**GAAATTCGCAAGTACCAGAAGAG**CACTGAGCTTTTGATTAGGAAGCTTCCATTCCAGAGATTGGTGCGTGAAATCGCTCAGGATTTCAAGACTGATTTGAGGTTTCAGAGCTCTGCTGTGTCTGCTCTCCAGGAAGCG**GCTGAGGCATACCTTGTTGG**

***FtAction***

NCBI Genbank ID: KC571237.1

**GGAAGTATAGCGTCTGGATTGGC**GGGTCGATTCTAGCTTCACTCAGCACTTTTCAACAGATGTGGATTTCCAAGAGCGAGTATGATGAATCCGGTCCA**GCAATCGTTCACCGCAAGTG**

***FtGAPDH***

NCBI Genbank ID: MK416199

**TGGAGCTGCTAAGGCTGTCG**GCAAGGTGCTACCATCTTTGAATGGAAAGTTGACTGGAATGTCATTCCGTGTCCCAACCGTTGATGTTTCTGTTGTTGACCTCACTGTCAGAATTGAAAAGCCAGCCTCC**TACGAGGACATCAAGAGTGCTATCA**

***FtEF-1α***

NCBI Genbank ID: MK430141

**GCTGCTGAGATGAACAAGAGGTC**CTTCAAGTACGCATGGGTGTTGGACAAGCTTAAGGCTGAGCGTGAGCGTGGAATCACCATTGAC**ATCGCGTTGTGGAAGTTTGAG**

***FtExpressed1***

NCBI Genbank ID: MK416200

**AGGCCAGTTCCTGCTGAATGTAATGC**CGAACTACATACAGATTATGGTGGTGCAGCAGTGAGATGGGGTCTAACCCATCACAAGGAAAGTGCGGCAGATTG**TTGCCAGGCTTGCTTGGATCAGGCTA**

***FtSAND***

NCBI Genbank ID: MK416201

**GACCCCCTTGCAGACAAAGCATTGGCA**ATCAAGACATGCAACCGCGTATG**CCAGTGGGTAAAAGACGTTGAGAACGAGA**

***FtCACS***

NCBI Genbank ID: MK430142

**AAGACAGTCAGTTTCGTGCCACCTGA**TGGATGAATTTGAATTGATGAAGTATCGCATCACTGAGGGTGTCATCTCCCCTTTCGATGTGTTGCCAACAATC**AAGGAGTTGGGTAGAACACGCATGGA**

***FtSTAR2***

NCBI Genbank ID: MK416202

**ATGAAGAGGCTCCGAGATGA**CATCAAGAGCCAGACGAATCTGGTAGAAACGGCACTTGCATTGGGTGCGACTCCAAGACAAGCGGTTCTTCAAAACAT**GAAACGGGCGCTAGTGATAG**
