# Supplementary figures and images for: Validation of reference genes for gene expression studies in tartary buckwheat (Fagopyrum tataricum Gaertn.) using quantitative real-time PCR
Source: PeerJ. 2019 Feb 26;7:e6522. doi: 10.7717/peerj.6522 (PMC6396815; doi:10.7717/peerj.6522)

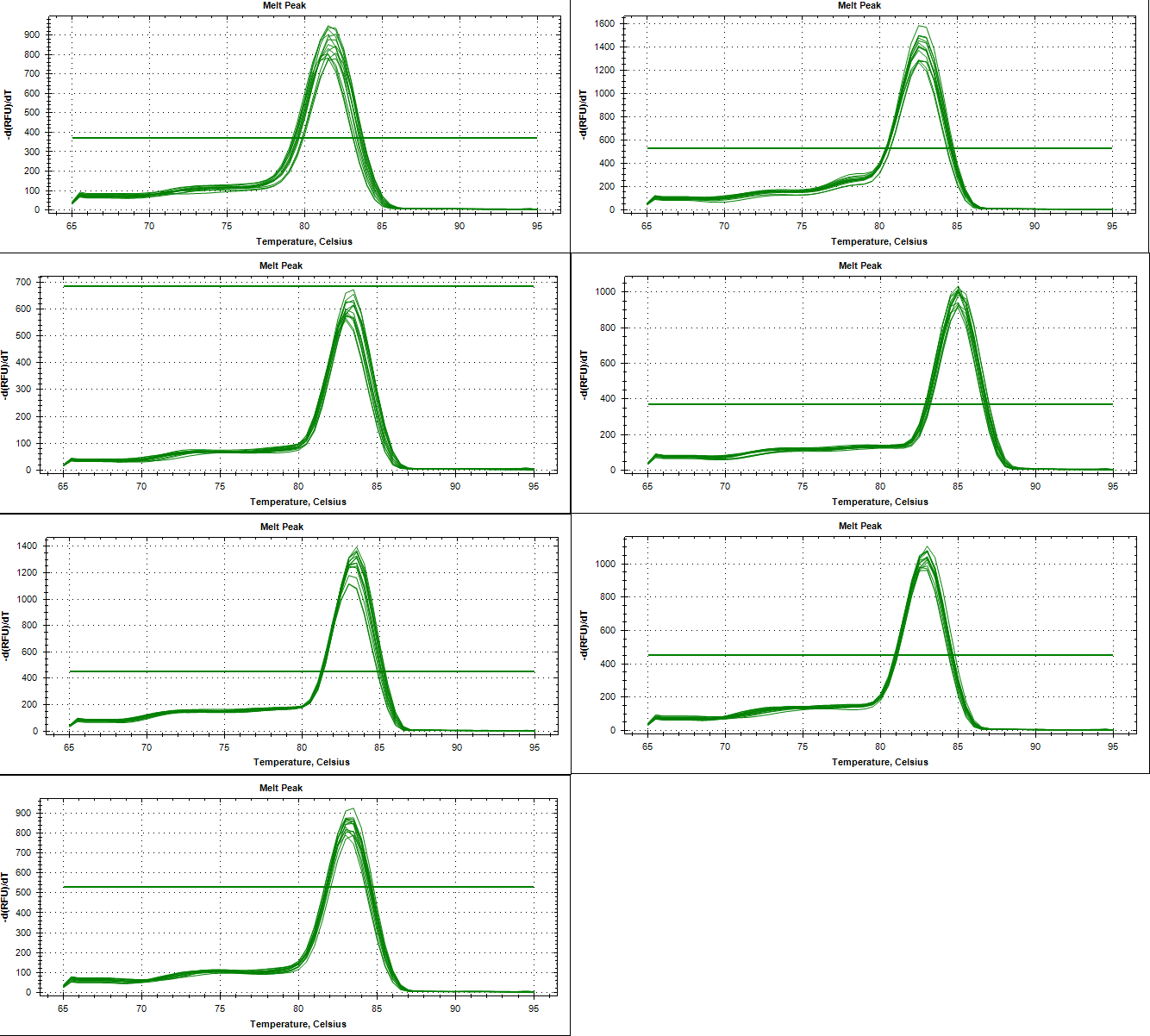


**Supplemental Fig. S1** Dissociation curves of the seven candidate reference genes

Supplement: Supplemental Information 2 [file peerj-07-6522-s002.docx]
