# Supplementary material for: Validation of reference genes for gene expression studies in tartary buckwheat (Fagopyrum tataricum Gaertn.) using quantitative real-time PCR
Source: PeerJ. 2019 Feb 26;7:e6522. doi: 10.7717/peerj.6522 (PMC6396815; doi:10.7717/peerj.6522)
Supplement: Supplemental Information 3 [file peerj-07-6522-s003.docx]

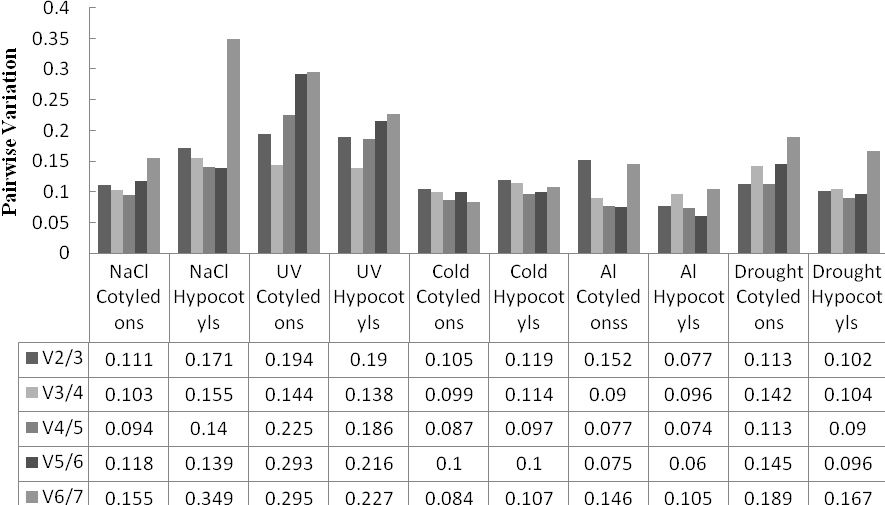


**Supplemental Fig. S2** Optimal number of reference genes required for accurate normalization in the hypocotyls and cotyledons of tartary buckwheat
