# Supplementary material for: Validation of reference genes for gene expression studies in tartary buckwheat (Fagopyrum tataricum Gaertn.) using quantitative real-time PCR
Source: PeerJ. 2019 Feb 26;7:e6522. doi: 10.7717/peerj.6522 (PMC6396815; doi:10.7717/peerj.6522)
Supplement: Supplemental Information 4 [file peerj-07-6522-s004.docx]

**Supplemental Table. S1** The stability of the expression of seven candidate reference genes in the hypocotyls and cotyledons of tartary buckwheat

| group | Rank | geNorm | | NormFinder | | BestKeeper | | |
| --- | --- | --- | --- | --- | --- | --- | --- | --- |
|  |  | Gene | Stability | Gene | Stability | Gene | SD[±Cq] | CV[%Cq] |
| UV  cotyledons | 1 | *FtCACS* | 0.502 | *FtCACS* | 0.134 | *FtH_3_* | 0.27 | 1.35 |
|  | 2 | *FtEF-1α* | 0.0.502 | *FtSAND* | 0.134 | *FtSAND* | 0.31 | 1.22 |
|  | 3 | *FtH_3_* | 0.590 | *FtH_3_* | 0.160 | *FtExpressed1* | 0.39 | 1.56 |
|  | 4 | *FtSAND* | 0.622 | *FtEF-1α* | 0.174 | *FtCACS* | 0.42 | 1.85 |
|  | 5 | *FtExpressed1* | 0.846 | *FtExpressed1* | 0.702 | *FtGAPDH* | 0.57 | 3.05 |
|  | 6 | *FtActin* | 1.171 | *FtActin* | 1.391 | *FtEF-1α* | 0.63 | 3.18 |
|  | 7 | *FtGAPDH* | 1.445 | *FtGAPDH* | 1.407 | *FtActin* | 0.90 | 4.26 |
| NaCl  cotyledons | 1 | *FtH_3_* | 0.392 | *FtCACS* | 0.179 | *FtCACS* | 0.13 | 0.59 |
|  | 2 | *FtExpressed1* | 0.392 | *FtEF-1α* | 0.283 | *FtH_3_* | 0.21 | 1.07 |
|  | 3 | *FtSAND* | 0.495 | *FtExpressed1* | 0.383 | *FtExpressed1* | 0.27 | 1.16 |
|  | 4 | *FtEF-1α* | 0.587 | *FtH_3_* | 0.423 | *FtSAND* | 0.30 | 1.20 |
|  | 5 | *FtGAPDH* | 0.661 | *FtSAND* | 0.446 | *FtEF-1α* | 0.57 | 3.22 |
|  | 6 | *FtCACS* | 0.753 | *FtGAPDH* | 0.482 | *FtGAPDH* | 0.87 | 5.15 |
|  | 7 | *FtActin* | 1.246 | *FtActin* | 1.686 | *FtActin* | 2.18 | 8.63 |
| Cold cotyledons | 1 | *FtH_3_* | 0.235 | *FtH_3_* | 0.081 | *FtSAND* | 0.18 | 0.73 |
|  | 2 | *FtCACS* | 0.235 | *FtCACS* | 0.128 | *FtCACS* | 0.19 | 0.88 |
|  | 3 | *FtActin* | 0.301 | *FtGAPDH* | 0.229 | *FtActin* | 0.28 | 1.26 |
|  | 4 | *FtSAND* | 0.362 | *FtActin* | 0.312 | *FtH_3_* | 0.29 | 1.48 |
|  | 5 | *FtGAPDH* | 0.409 | *FtSAND* | 0.322 | *FtGAPDH* | 0.33 | 2.03 |
|  | 6 | *FtExpressed1* | 0.489 | *FtExpressed1* | 0.341 | *FtExpressed1* | 0.49 | 2.11 |
|  | 7 | *FtEF-1α* | 0.535 | *FtEF-1α* | 0.392 | *FtEF-1α* | 0.53 | 3.13 |
| PEG  cotyledons | 1 | *FtCACS* | 0.221 | *FtCACS* | 0.087 | *FtCACS* | 0.23 | 0.99 |
|  | 2 | *FtH_3_* | 0.221 | *FtH_3_* | 0.128 | *FtSAND* | 0.24 | 0.98 |
|  | 3 | *FtSAND* | 0.310 | *FtSAND* | 0.216 | *FtH_3_* | 0.31 | 1.56 |
|  | 4 | *FtEF-1α* | 0.451 | *FtExpressed1* | 0.236 | *FtExpressed1* | 0.35 | 1.45 |
|  | 5 | *FtExpressed1* | 0.523 | *FtEF-1α* | 0.289 | *FtEF-1α* | 0.54 | 2.95 |
|  | 6 | *FtGAPDH* | 0.657 | *FtGAPDH* | 0.705 | *FtGAPDH* | 0.76 | 4.66 |
|  | 7 | *FtActin* | 0.859 | *FtActin* | 0.909 | *FtActin* | 0.97 | 4.30 |
| PEG  hypocotyls | 1 | *FtH_3_* | 0.141 | *FtCACS* | 0.049 | *FtActin* | 0.27 | 1.14 |
|  | 2 | *FtExpressed* | 0.141 | *FtH_3_* | 0.095 | *FtSAND* | 0.32 | 1.25 |
|  | 3 | *FtEF-1α* | 0.255 | *FtExpressed1* | 0.162 | *FtH_3_* | 0.32 | 1.58 |
|  | 4 | *1 FtCACS* | 0.346 | *FtSAND* | 0.269 | *FtCACS* | 0.45 | 2.08 |
|  | 5 | *FtSAND* | 0.398 | *FtEF-1α* | 0.366 | *FtExpressed1* | 0.51 | 2.17 |
|  | 6 | *FtGAPDH* | 0.464 | *FtActin* | 0.370 | *FtEF-1α* | 0.75 | 4.39 |
|  | 7 | *FtActin* | 0.494 | *FtGAPDH* | 0.805 | *FtGAPDH* | 0.76 | 4.43 |
| Cold hypocotyls | 1 | *FtCACS* | 0.207 | *FtH_3_* | 0.072 | *FtCACS* | 0.12 | 0.53 |
|  | 2 | *FtH_3_* | 0.207 | *FtCACS* | 0.072 | *FtH_3_* | 0.18 | 0.90 |
|  | 3 | *FtActin* | 0.311 | *FtGAPDH* | 0.244 | *FtActin* | 0.32 | 1.37 |
|  | 4 | *FtGAPDH* | 0.398 | *FtActin 1* | 0.247 | *FtGAPDH* | 0.33 | 1.95 |
|  | 5 | *FtEF-1α* | 0.455 | *FtEF-1α* | 0.311 | *FtEF-1α* | 0.35 | 2.08 |
|  | 6 | *FtExpressed1* | 0.521 | *FtExpressed* | 0.468 | *FtExpressed1* | 0.43 | 1.81 |
|  | 7 | *FtSAND* | 0.595 | *FtSAND* | 0.508 | *FtSAND* | 0.55 | 2.02 |
| NaCl  hypocotyls | 1 | *FtCACS* | 0.318 | *FtCACS* | 0.110 | *FtExpressed1* | 0.15 | 0.60 |
|  | 2 | *FtExpressed1* | 0.318 | *FtExpressed1* | 0.166 | *FtSAND* | 0.19 | 0.71 |
|  | 3 | *FtH_3_* | 0.352 | *FtH_3_* | 0.212 | *FtH_3_* | 0.19 | 0.90 |
|  | 4 | *FtSAND* | 0.400 | *FtSAND* | 0.257 | *FtEF-1α* | 0.43 | 2.44 |
|  | 5 | *FtActin* | 0.450 | *FtActin* | 0.376 | *FtActin* | 0.44 | 1.98 |
|  | 6 | *FtEF-1α* | 0.547 | *FtEF-1α* | 0.478 | *FtGAPDH* | 0.58 | 3.30 |
|  | 7 | *FtGAPDH* | 0.718 | *FtGAPDH* | 0.745 | *FtCACS* | 0.65 | 2.76 |
| UV  hypocotyls | 1 | *FtH _3_* | 0.178 | *FtExpressed1* | 0.195 | *FtActin* | 0.24 | 0.99 |
|  | 2 | *FtActin* | 0.178 | *FtH_3_* | 0.469 | *FtH_3_* | 0.45 | 2.12 |
|  | 3 | *FtExpressed1* | 0.443 | *FtActin* | 0.471 | *FtCACS* | 0.70 | 2.97 |
|  | 4 | *FtCACS* | 0.529 | *FtCACS* | 0.498 | *FtExpressed1* | 0.85 | 3.32 |
|  | 5 | *FtGAPDH* | 0.705 | *FtGAPDH* | 0.592 | *FtSAND* | 1.01 | 3.82 |
|  | 6 | *FtSAND* | 0.929 | *FtSAND* | 0.691 | *FtEF-1α* | 1.56 | 8.14 |
|  | 7 | *FtEF-1α* | 1.14 | *FtEF-1α* | 1.085 | *FtGAPDH* | 1.93 | 9.70 |
